# Evaluation of Digital Interventions for Physical Activity Promotion: Protocol for a Scoping Review

**DOI:** 10.2196/35332

**Published:** 2022-03-03

**Authors:** Karina Karolina De Santis, Tina Jahnel, Lea Mergenthal, Hajo Zeeb, Katja Matthias

**Affiliations:** 1 Department of Prevention and Evaluation Leibniz Institute for Prevention Research and Epidemiology – BIPS Bremen Germany; 2 Faculty 11 Human and Health Sciences University of Bremen Bremen Germany; 3 Faculty of Electrical Engineering and Computer Science University of Applied Science Stralsund Stralsund Germany

**Keywords:** evaluation, digital intervention, physical activity promotion, scoping review, digital health

## Abstract

**Background:**

Digital interventions (DIs) could support physical activity (PA) promotion, according to recent reviews. However, it remains unclear if and how DIs for PA promotion are evaluated; thus, it is unclear if they support behavior change in real-world settings. A mapping of evidence from published reviews is required to focus on the evaluation of DIs for PA promotion.

**Objective:**

The aim of our study is to investigate evaluation strategies for any outcome in the context of DIs for PA promotion by conducting a scoping review of published reviews.

**Methods:**

Our scoping review adheres to the PRISMA-ScR (Preferred Reporting Items for Systematic Reviews and Meta-Analyses extension for Scoping Reviews) guidelines. The information sources include bibliographic databases (MEDLINE, PsycINFO, and CINAHL) and the bibliographies of the selected studies. The electronic search strategy was developed and conducted in collaboration with an experienced database specialist. The electronic search was conducted in English with no limits up to March 19, 2021, for sources with the terms *digital intervention AND evaluation AND physical activity* in titles or abstracts. After deduplication, 300 reviews selected from 4912 search results were assessed for eligibility by 2 authors working independently. The inclusion criteria were (1) healthy or clinical samples (population), (2) DIs for PA promotion (intervention), (3) comparisons to any other intervention or no intervention (comparison), (4) evaluation strategies (methods, results, or frameworks) for any outcome in the context of DIs for PA promotion (outcome), and (5) any published review (study type). According to the consensus reached during a discussion, 40 reviews met the inclusion criteria—36 from the electronic search and 4 from the manual search of the bibliographies of the 36 reviews. All reviews reported the evaluation strategies for any outcomes in the context of DIs for PA promotion in healthy or clinical samples. Data coding and the quality appraisal of systematic reviews are currently being performed independently by 2 authors.

**Results:**

Our scoping review includes data from 40 published reviews (1 rapid review, 9 scoping reviews, and 30 systematic reviews). The focus of data coding is on evaluation strategies in the context of DIs for PA promotion and on the critical appraisal of the included systematic reviews. The final consensus regarding all data is expected in early 2022.

**Conclusions:**

Interventions for PA promotion that are supported by digital technologies require evaluation to ensure their efficacy in real-world settings. Our scoping review is needed because it addresses novel objectives that focus on such evaluations and are not answered in the published reviews identified in our search. The evaluation strategies addressing DIs for PA promotion will be mapped to synthesize the results that have been reported in published reviews so far.

**International Registered Report Identifier (IRRID):**

DERR1-10.2196/35332

## Introduction

The field of digital public health is rapidly developing [1]. Digitization is likely to have a major impact on therapy in the future, and it already increasingly contributes to prevention and health promotion [2]. Interventions supported by modern technologies (ie, digital interventions [DIs]) are enormously popular in the context of healthy lifestyle and behavior change, including physical activity (PA) promotion [3,4]. A number of challenges exist in this rapidly developing field, including the gap between clinical or preventive interests and commercial interests [5], the ethics of data storage and usage [6], and development issues (theoretical and evidence-based foundations of new DIs) [7].

One key question in this new field of research is if DIs truly work in any health context. In light of the long history of evidence-based medicine with guidelines on how to assess the effectiveness of nondigital health interventions, comprehensive guidelines on the systematic evaluation of DIs are still scarce. Evaluation is also important for justifying and informing policy, program, and funding decisions. Although initial evaluation criteria and frameworks have been proposed for DIs, this preliminary work lacks guidance as to when and to which degree these criteria should be applied [8-10]. For example, as already pointed out in 2015 [11], it remains unclear if and how DIs for PA promotion are evaluated. Evaluation in this context is essential for understanding if DIs support behavior change in real-world settings, so that the sustainable, effective, and efficient use of DIs can be achieved [12,13]. However, real-world DIs are complex and difficult to evaluate. Among others, the challenges of evaluating DIs include contextual factors, such as settings, target populations, intervention functions, or intended outcomes; as well as organizational, political, or resourcing factors. Some of the practical challenges in conducting evaluations include using appropriate evaluation methods and tools, understanding what counts as evidence, and understanding how such evidence is applied and interpreted [14-16]. Therefore, a review of the literature is required to focus on evaluation methods in the context of DIs for PA promotion.

According to a PubMed search, 155 reviews with the terms *digital AND physical activity* have already been published up to November 17, 2021 (including 55 reviews in 2021 alone). Due to the high number of potentially relevant reviews that have already been published on this topic, the mapping of existing evidence is required to investigate if and how the evaluation of DIs for PA promotion was addressed in such publications. Mapping is important for identifying any evidence gaps that could be addressed in future reviews of primary studies.

**Figure 1:**
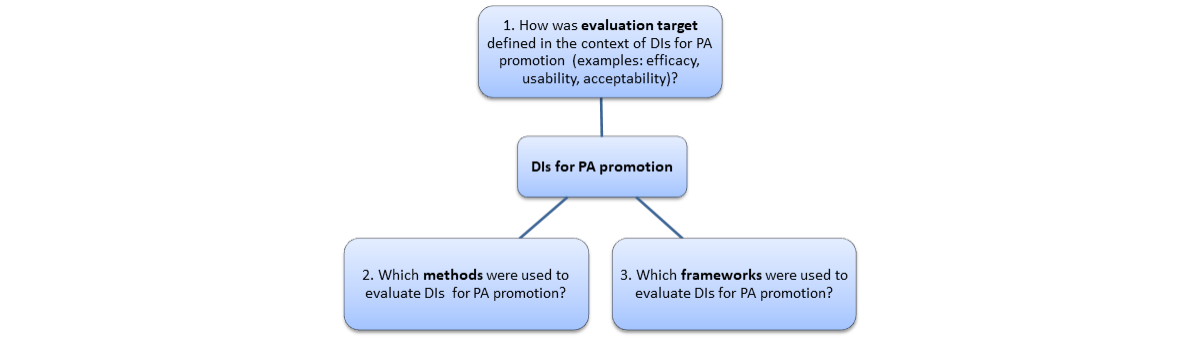
Objectives of this scoping review. DI: digital intervention; PA: physical activity.

The aim of this study is to investigate evaluation strategies for any outcome in the context of DIs for PA promotion by conducting a scoping review of published reviews. The three main objectives of this scoping review address the evaluation target, methods, and theoretical frameworks (Figure 1).

## Methods

### Study Design

Our study uses a scoping review design. The study adheres to the PRISMA-ScR (Preferred Reporting Items for Systematic Reviews and Meta-Analyses extension for Scoping Reviews) guidelines [17]. The PRISMA-ScR checklist will be reported in an appendix once the scoping review is complete.

### Protocol and Registration

The study was registered at the Center for Open Science [18] and was planned to include the following two parts: (1) an overview (review) of reviews and (2) a scoping review of primary studies. This protocol addresses only part 1 of the planned study. The need for part 2 will be established once the results of part 1 are available. In contrast to our registration [18], the study will be performed as a scoping review of reviews due to the availability of appropriate guidance (PRISMA-ScR [17]). Furthermore, our study has a broader focus on evaluation methods relative to that of overviews of reviews that typically address specific outcomes of interventions in the health context.

The electronic literature search was conducted on March 19, 2021, prior to study registration on May 3, 2021 [18]. The results were screened by 1 author for the presence of other overviews or scoping reviews addressing the same aims as those planned in our study. This step was necessary to prevent any research waste. According to meta-research [19-21], many reviews of interventions in the health context are redundant because they either address the same aims as those addressed by other existing reviews or cite the same primary studies. This problem is so extensive that some reviews do not include any unique primary studies that are not cited in other reviews, and there are as many reviews as or even more reviews than there are primary studies in some fields [19-21]. The procedure of checking if a new review is required prior to study registration may be especially necessary in rapidly developing fields, such as digitally supported interventions, or when addressing commonly investigated outcomes, such as PA promotion.

### Eligibility Criteria

The eligibility criteria for our scoping review are based on the Population, Intervention, Comparison, Outcome, and Study Type (PICOS) criteria (Textbox 1). Our review has a methods focus and thus targets any outcome.

**Eligibility criteria for the scoping review.**

**Inclusion criteria**

1. Population: human samples of any age (children or adults) and health status (healthy or clinical)
2. Intervention: digital interventions for physical activity promotion as a primary outcome
3. Comparison: comparisons to any other intervention or no intervention
4. Outcome: evaluation of a digital intervention that is planned or performed by using any method for any target
5. Study type: any review (systematic, scoping, rapid, narrative, overview, or other)
6. Publication status: published in a peer-reviewed journal
7. Publication language: English or German
8. Access to the full texts of studies selected for data coding

**Exclusion criteria**

1. Nonhuman studies
2. Digital interventions for physical activity promotion are not applied or are not the primary intervention (included as a control to or part of another intervention)
3. Evaluation of digital interventions is not addressed (not planned or not performed)
4. Other study type: primary study, comment, correction, letter, editorial, or protocol
5. Other publication status: conference paper, unpublished report, thesis, or book
6. Language other than English or German
7. No access to the full texts of studies selected for data coding

### Information Sources

The information sources for the scoping review include bibliographic databases (MEDLINE, PsycINFO, and CINAHL) as well as the bibliographies of the selected studies. These databases were chosen because they delivered the most relevant studies in our other searches for DIs in the context of public health.

### Search

The electronic search strategy was developed iteratively by the team in consultation with a professional librarian. The search terms and corresponding MeSH (Medical Subject Headings) terms were derived to address the following three main search topics: (1) DIs (with a mobile app), (2) evaluation, and (3) PA. The full search strategy will be reported in an appendix once the scoping review is complete. A summary of the electronic search and its outcomes is shown in Table 1. In addition, the bibliographies of the included studies were manually screened for additional relevant sources.

**Table 1:** Summary of the electronic search strategy^a^.

Databases (time frame)	Search strategy summary (search terms)	Studies (N=8272), n
MEDLINE through Ovid (from inception through to March 19, 2021)	Title OR abstract (*mobile application AND evaluation AND physical activity*)	4776
PsycINFO through Ovid (from inception through to March 19, 2021)	Title OR abstract (*mobile application AND evaluation AND physical activity*)	1157
CINAHL through EBSCO (from inception through to March 19, 2021)	Title OR abstract (*mobile application AND evaluation AND physical activity*)	2339

^a^The electronic search was conducted in English with no limits by a team assistant on March 19, 2021.

### Selection of Sources of Evidence

The electronic search results (8272 studies) were stored in EndNote X9 (Clarivate). Following the removal of duplicates in EndNote, 4912 remaining studies were divided into 2 groups (reviews or nonreviews) by using the *smart groups* function in EndNote. All identified reviews (300/4912, 6.11%) were exported into a new EndNote library and divided into the following five groups, depending on review type, by using the *smart groups* function: (1) overview of reviews, (2) rapid review, (3) scoping review, (4) narrative review, and (5) systematic review.

**Figure 2:**
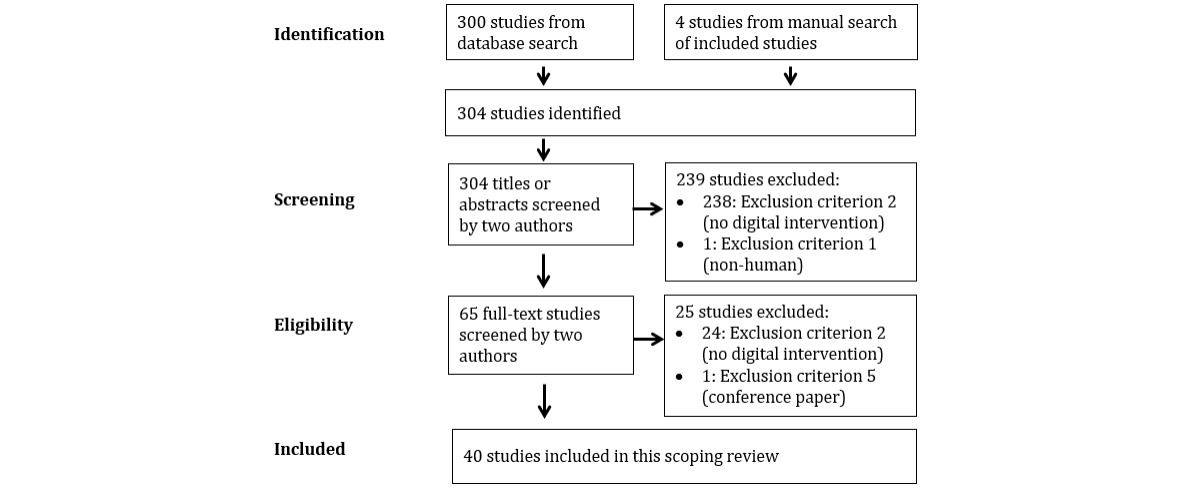
Study selection (PRISMA [Preferred Reporting Items for Systematic Reviews and Meta-Analyses] flowchart).

Study selection was conducted in 3 steps. First, 2 authors independently screened all titles and abstracts in each smart group for inclusion and reached consensus during a discussion. Second, 2 authors independently screened the studies selected for the full-text inspection and reached consensus during a discussion. Third, once the final study selection from the electronic search was completed, 2 authors manually screened the bibliographies of the included studies for additional relevant sources and reached consensus during a discussion. The outcomes of the study selection are summarized in Table 2 and Figure 2. The complete list of included and excluded studies will be reported in an appendix once the scoping review is complete.

**Table 2:** Summary of study selection.

Review type^a^				Studies, n		Exclusion^b^
**Reviews from electronic search (n=300)**						
	**Overview**			3		N/A^c^
		Excluded based on title or abstract	3		Exclusion criterion 2	
		Excluded based on full text	0		N/A	
		Included	0		N/A	
	**Rapid review**			4		N/A
		Excluded based on title or abstract	3		Exclusion criterion 2	
		Excluded based on full text	0		N/A	
		Included	1		N/A	
	**Scoping review**			21		N/A
		Excluded based on title or abstract	1		Exclusion criterion 1	
		Excluded based on title or abstract	10		Exclusion criterion 2	
		Excluded based on full text	1		Exclusion criterion 2	
		Included	9		N/A	
	**Narrative review**			51		N/A
		Excluded based on title or abstract	45		Exclusion criterion 2	
		Excluded based on full text	6		Exclusion criterion 2	
		Included	0		N/A	
	**Systematic review**			221		N/A
		Excluded based on title or abstract	177		Exclusion criterion 2	
		Excluded based on full text	17		Exclusion criterion 2	
		Excluded based on full text	1		Exclusion criterion 5	
		Included	26		N/A	
**Reviews from manual search (n=4)**						
	**Systematic review**			4		N/A
		Included from the manual search of the bibliographies of all included reviews	4		N/A	

^a^Review type was established based on the information in titles or abstracts. A total of 40 reviews were included from the electronic and manual searches.

^b^Exclusion criteria are shown in Textbox 1.

^c^N/A: not applicable.

### Data Charting

A form for coding and capturing all data was self-developed in Microsoft Excel and calibrated within the team. Two authors will code all data independently and reach consensus during a discussion. Data coding is currently in progress.

### Data Items

Data items that will be coded in the scoping review are reported in Textbox 2. These items were chosen to address the objectives of the scoping review (Figure 1). All data will be reported in an appendix once the scoping review is complete.

In addition to the definitions provided in Textbox 2, the following operationalization definitions were used to improve the reliability and validity of study selection and data coding in the scoping review:

1. A *healthy* population was defined as samples without acute or chronic illnesses, but this could include samples at risk for various clinical illnesses.
2. *Digital intervention* was defined as an intervention delivered or supported by digital tools. *Digital tools* were defined as any digitally supported technologies for automated and continuous self-monitoring and feedback. This includes smartphone apps, activity trackers, and web-based software but excludes digital tools, such as pedometers and accelerometers, that do not offer tracked measures or feedback over time [22]. Pedometers or accelerometers were included as part of DIs or with other digital tools in the minority of primary studies in some reviews. Reviews were excluded if all or the majority of their primary studies used only pedometers or accelerometers.
3. *PA promotion* was defined as any primary outcome focusing on PA promotion. Reviews were excluded if PA promotion was assessed as part of a healthy lifestyle or as a secondary outcome for the management of weight, blood sugar, or sports injuries; for balance and mobility training following surgeries; or for such training in the management of neurological disorders.

**Data items in this scoping review.**

**Bibliographic information**

- First author
- Year of publication
- Region of corresponding author (continent)
- Study title
- Study aim according to study authors
- Funding sources and conflicts of interest according to study authors

**Population**

- Population by health status (healthy individuals or individuals with clinical diagnoses)
- Population by diagnosis (none or diagnosis name)
- Population by diagnosis type (none, any, mental, neurological, or somatic)
- Population by age (any age; minors aged up to 18 years; or adults aged 18 years or older, including specific subgroups [eg, older adults])

**Intervention**

- Any digital intervention

**Comparison**

- Comparison (any, independent control group with another intervention, or baseline in pre-post studies without control groups)

**Outcome**

- Any outcome in the context of physical activity promotion
- Outcome focus (general fitness, mobility, or other)

**Study (review) type**

- Review type (rapid, scoping, or systematic)
- Primary studies in review (number)
- Published primary studies in review (number)
- Unique published studies that do not overlap with primary studies in other reviews (number)
- Primary study design in review (only randomized controlled trials [RCTs] or any designs, including RCTs and non-RCTs)

**Evaluation**

- Evaluation target (user outcomes [eg, efficacy, usability, acceptability, or tool performance or validation])
- Evaluation method (objective automated data from the tools, scales, or tests or the validation of tool data vs another method)
- Theory framework type (not reported or framework name)
- Theory framework description according to study authors (eg, frameworks used in tool development)
- Requirements for the efficacy of digital interventions according to study authors (eg, engagement with the tool)
- Evidence gaps according to study authors (recommendations for future research, limitations, and conclusions)

### Critical Appraisal of Individual Sources of Evidence

The critical appraisal will be conducted only for the systematic reviews by using a tool that was specifically developed for such reviews (A Measurement Tool to Assess Systematic Reviews, Version 2 [AMSTAR2]) [23]. AMSTAR2 has acceptable psychometric properties and is an appropriate tool for appraising systematic reviews of interventions in the health context [23,24]. The tool includes 16 items that need to be rated to derive the overall confidence rating for the results of a systematic review (critically low, low, moderate, or high) [23]. All 16 items will be rated according to the AMSTAR2 scoring guidelines [23]. The overall confidence rating will be derived for each systematic review based on a combination of scores for 7 critical items and 9 noncritical items, in accordance with the AMSTAR2 guidelines [23]. In general, critically low ratings are assigned if at least 2 critical items are not fulfilled (rated as “no”) on AMSTAR2.

A form for appraising systematic reviews with AMSTAR2 was self-developed in Microsoft Excel. Two authors will appraise all systematic reviews independently and reach consensus during a discussion. The appraisals will be performed in 2 phases by both authors.

During phase 1, the systematic reviews with critically low confidence ratings will be identified. This will be done by using 2 AMSTAR2 items (item 2: presence of a review protocol; item 7: presence of a list of excluded studies). Systematic reviews that do not fulfill both of these items will receive critically low confidence ratings. These two items address the most common limitations in systematic reviews of interventions in the health context [20], and they were selected according to a fast and frugal decision tree for the critical appraisal of systematic reviews [25].

During phase 2, the systematic reviews that fulfilled at least 1 of the 2 items in phase 1 (item 2 or 7) will be rated by using all 16 AMSTAR2 items. This will be done to identify further systematic reviews with critically low confidence ratings and other reviews with low, moderate, or high confidence ratings.

Once consensus is reached, the final overall confidence ratings for each systematic review derived from AMSTAR2 will be reported in an appendix. The appraisal procedure is currently in progress.

In addition to deriving the overall confidence ratings, the AMSTAR2 scores will be used for 2 meta-research studies. Both studies will be performed because the overall confidence ratings on AMSTAR2 alone poorly discriminate among systematic reviews of various interventions in the health context [20].

The first meta-research study will be performed in addition to our original registration [18]. This study will address the following two aims: (1) to identify common strengths and weaknesses in systematic reviews of DIs for PA promotion and (2) to assess the stability of the overall confidence ratings. To investigate both aims, all systematic reviews appraised with the two AMSTAR2 items in phase 1 will be appraised with all 16 AMSTAR2 items. Two authors will appraise all systematic reviews independently and reach consensus during a discussion. To address aim 1, the scores on the individual AMSTAR2 items for each of the 30 systematic reviews will be presented on a bar graph to visualize the strengths (fulfilled items rated as “yes” or “partial yes”) and the weaknesses (not fulfilled items rated as “no”) in each review. To address aim 2, the outcomes of appraisals involving 2 AMSTAR2 items and those involving 16 AMSTAR2 items will be compared descriptively according to the overall rating correctness and the total appraisal time. Furthermore, the “yes” or “yes + partial yes” ratings will be expressed as percentage scores out of all 16 ratings assigned to each systematic review in accordance with methods described elsewhere [26]. Such percentage scores for “yes” or “yes + partial yes” ratings will be compared between 2 groups of systematic reviews, which will be based on the reviews’ overall confidence ratings (critically low and low vs moderate and high). The comparisons will be computed in IBM-SPSS 24 (IBM Corporation) and reported as odds ratios with 95% CIs for the nominal variables or as mean difference scores with 95% CIs for the continuous variables. Finally, the overall confidence ratings derived from different combinations of critical items will be descriptively compared.

The second meta-research study will be performed in accordance with the plan in our original registration [18]. The aim of the second meta-research study is to compare the outcomes of AMSTAR2 appraisals by using the original scoring guidelines [23] and the revised scoring guidelines proposed by us. Two authors will appraise the same systematic reviews independently; one will use the original scoring guidelines, and one will use the revised scoring guidelines. The overall confidence ratings will be graphically summarized and descriptively compared. This will be done to identify the sources of similarities and discrepancies between the outcomes of both scoring methods and to test the usefulness of the revised scoring guidelines for appraising systematic reviews.

### Synthesis of Results

Studies will be grouped according to their designs. The coded data will be synthesized either by using descriptive statistics (relative frequencies) or narratively within each group. If applicable, evidence maps [27] will be used to visualize the results according to the three objectives of the scoping review (Figure 1). The overall confidence ratings for all systematic reviews will be graphically synthesized by using a bar graph to visualize the outcomes of the critical appraisal.

## Results

### Included Studies

Our electronic search identified 6.11% (300/4912) studies designated as reviews of any type in titles or abstracts. Of the 300 reviews, 36 (12%) met the inclusion criteria (Textbox 1) for the scoping review. An additional 4 reviews were selected following a manual search of the bibliographies of the 36 included reviews. Thus, 40 reviews were included in the scoping review (Figure 2). Of the 40 reviews, 1 (2.5%) was a rapid review, 9 (22.5%) were scoping reviews, and 30 (75%) were systematic reviews (Table 2).

### Further Results

Data coding and the critical appraisal of systematic reviews are currently in progress and are expected to be completed in early 2022.

## Discussion

### Principal Results

Our electronic search revealed that 300 reviews indexed in 3 bibliographic databases have already been published on interventions supported by digital technologies and designed for PA. Thus, to prevent research waste resulting from the contribution of another review of primary studies, our scoping review will synthesize the findings of such published reviews. Among the 300 reviews, we identified 40 reviews that specifically focused on evaluation strategies in the context of DIs for PA promotion (36 from the electronic search and 4 from the manual search of the bibliographies of the 36 included reviews). According to our preliminary findings, our scoping review is needed because it addresses novel objectives that focus on evaluation strategies in the context of DIs for PA promotion.

### Interest in DIs for PA Promotion

The large number of reviews on digitization and PA highlights 2 important issues so far. First, the academic field of DIs for the core aspects of public health (prevention and health promotion) is rapidly developing [1]. This development is probably related to the general technological progress [4] and economic interest in the digitization of health [5]. However, the digitization of health is also associated with various challenges, such as access to digital tools, digital health competence, and ethical issues related to data storage and usage [28]. Interestingly, although digital technologies for PA are enormously popular [3], it remains unclear if they work (ie, if they promote PA). Second, there is a need to carefully inspect the published literature on digitization and PA promotion before conducting a new review that may be redundant and may contribute to research waste. Our scoping review of other reviews will help to identify evidence gaps and possible research questions that have not been addressed in the academic literature so far. Such evidence gaps will be used to determine if a new scoping review or systematic review of primary studies is required in this rapidly developing field.

### Limitations

Although the data coding and appraisal are still ongoing, 3 main limitations have already been identified in the scoping review. First, the development of the search strategy was difficult due to the heterogeneous terminology used in the field of DIs. Professional assistance with the development of the search syntax for bibliographic databases was an essential requirement for determining the validity of our search. Our search syntax was calibrated and extensively pretested, and the search was conducted under the supervision of an experienced librarian who specialized in bibliographic databases. Although a large number of relevant sources suggests that the search was valid, we acknowledge that additional search terms could have been used to identify further sources. Second, we searched only 3 electronic databases (MEDLINE, PsycINFO, and CINAHL). The pilot searches for reviews on DIs for PA promotion in the Cochrane Library and Scopus did not identify any additional relevant reviews. However, it cannot be ruled out that additional sources are available on other international databases. Third, the careful operationalization of definitions is required for study selection and data coding because this research field is young and is evolving. Thus, the definitions of some PICOS criteria (Textbox 1) had to be expanded to improve the reliability and validity of the study selection and data coding processes that were conducted after the pilot assessment of scoping reviews. Finally, the data sources for our scoping review (other published reviews) may not necessarily focus on the evaluation of DIs for PA promotion. Although this cannot be ruled out, our search strategy indeed included the term *evaluation*, meaning that all of the studies identified in the search included this term in their titles, abstracts, or keywords. Thus, the scoping review can systematically collate the information about the evaluation of DIs for PA promotion from other reviews to identify any evidence gaps that could be addressed in future reviews of primary studies. In general, our experiences so far highlight the need for high-quality documentation and the reporting of definitions in this relatively new and dynamically developing field.

### Conclusions

Interventions for PA promotion supported by digital technologies require evaluation to ensure their efficacy in real-world settings. Our scoping review is needed because it addresses novel objectives that focus on such evaluations and are not answered in the published reviews identified in our search. The evaluation strategies addressing DIs for PA promotion will be mapped to synthesize the results that have been reported in published reviews so far.

## Abbreviations

AMSTAR2: A Measurement Tool to Assess Systematic Reviews, Version 2
DI: digital intervention
MeSH: Medical Subject Headings
PA: physical activity
PICOS: Population, Intervention, Comparison, Outcome, Study Type
PRISMA-ScR: Preferred Reporting Items for Systematic Reviews and Meta-Analyses extension for Scoping Reviews
